# The state of global surgery assessment and data collection tools: A scoping review

**DOI:** 10.1002/wjs.12380

**Published:** 2024-10-28

**Authors:** Kevin Gianaris, Brooke Stephanian, Sabin Karki, Shailvi Gupta, Amila Ratnayake, Adam L. Kushner, Reinou S. Groen

**Affiliations:** ^1^ Indiana University School of Medicine Indianapolis Indiana USA; ^2^ University of Maryland Medical Center Baltimore Maryland USA; ^3^ Military Hospital Narahenpita Colombo Sri Lanka; ^4^ Surgeons OverSeas New York New York USA; ^5^ Alaska Native Medical Center Anchorage Alaska USA

**Keywords:** global surgery

## Abstract

**Background:**

There has been a proliferation of global surgery assessment tools designed for use in low‐ and middle‐income countries. This scoping review sought to categorize and organize the breadth of global surgery assessment tools in the literature.

**Methods:**

The search was conducted using PubMed from October 2022 to April 2023 according to PRISMA extension for scoping review guidelines. The search terms were ((“global surgery”[All Fields]) AND (“assessment”[All Fields]) OR (data collection)). Only tools published in English that detailed surgical assessment tools designed for low‐ and middle‐income countries were included.

**Results:**

The search resulted in 963 papers and 46 texts described unique tools that were included for the final review. Of these, 30 (65%) tools were quantitative, 1 (2%) qualitative, and 15 (33%) employed mixed‐methods. 25 (54%) tools evaluated surgery in general, whereas 21 (46%) were focused on various surgical subspecialties. Qualitatively, major themes among the tools were noted. There was significant overlap of many tools.

**Conclusions:**

Nonspecialty surgery was represented more than any specialty surgery and many specialties had little or no representation in the literature. Ideally, local leadership should be involved in surgical assessment tools. Different methodologies, such as checklists and observational studies, aimed to target varying aspects of surgery and had distinct strengths and weaknesses. Further efforts should focus on expanding tools in neglected specialties.

## 1 INTRODUCTION

The UN Sustainable Development Goals support the adoption of robust monitoring of healthcare services at the national scale across both public and private sectors to establish measures of accountability and facilitate data comparison.^1^ However, significant gaps remain in health data globally today. Many countries still lack up‐to‐date information on healthcare availability and readiness, making it difficult to assess and improve on gaps in services provided. Access to quality healthcare services is a fundamental function of any health system and encompasses availability, affordability, and acceptability and it is necessary to target the data collection and assessment systems as a means for improvement in these areas. To address this, in 2023 the World Health Organization (WHO) adopted a mandate on “Integrated emergency, critical and operative care for universal health coverage and protection from health emergencies.^2^” The mandate examines the holistic paradigm of healthcare delivery and rolls together emergency, critical, and operative care to develop a coordinated approach for services outside of primary care. It draws a distinction between primary care delivery and development and distances itself from the previous model of global surgery as a separate entity that should be approached individually as outlined in the Lancet Commission on Global Surgery.^3^

Within this paradigm, surgery specifically has been shown to be a cost‐effective and necessary part of healthcare delivery in low‐, middle‐, and high‐income settings.^4^ The surgical aspects of care are defined to include the American College of Surgeons' five phases of surgical care: preoperative, perioperative, intraoperative, postoperative, and post‐discharge.^5^ Over the years, a variety of tools have been developed to measure surgical needs and capacities and to collect data in low‐ and middle‐income countries (LMICs). Because there are unique challenges to be faced with surgery data collection in LMICs, tools specified for that purpose have been developed.

Global surgery assessment tools are instruments used to evaluate the availability, accessibility, need, and at times quality of surgical care in LMICs. These tools assess the entire spectrum of the surgical system, including infrastructure, human resources, equipment, supplies, and processes. The aim of these tools is to identify gaps and challenges in surgical care and, ultimately, inform the development of targeted interventions to improve access and quality of care.

The growth of academic global surgery as a field has resulted in the development, proliferation, and publication of many global surgery assessment tools in the literature. Although some reviews have attempted to categorize and organize the available global surgery tools for trauma and pediatric surgery specifically, this scoping review seeks to synthesize and identify the breadth of published academic global surgery assessment tools.^6^, ^7^, ^8^

## 2 METHODS

The search was conducted using PubMed from October 2022 to April 2023 according to PRISMA extension for scoping review guidelines. The search terms were ((“global surgery”[All Fields]) AND (“assessment”[All Fields]) OR (data collection)). Covidence software was used to organize the papers. Initial title and abstract screening were performed by three independent authors and compared to come to a consensus of which papers to undergo full‐text review. Once included, papers underwent full‐text review by three independent authors and a consensus was reached to include or exclude each paper. The papers were then categorized as quantitative, qualitative, or mixed‐methods. The number of citations for each tool was roughly estimated using the initial publication of each tool using the citations tool on PubMed.

### 2.1 Inclusion and exclusion criteria

Papers which were included detailed the use or development of a unique surgical assessment or data collection tool designed for or used in LMICs. Only papers published in English were included in this review. This includes surveys, interview guides, checklists, observation guides, and unique methods of data collection or analysis, among other types of tools. Only one paper per tool was included and a representative published manuscript was cited to represent each tool. The included publications were then forward and reverse searched for relevant references that included unique surgical tools. Reviews, meta‐analyses, and consensus reports were excluded from the study, but their references were forward and reverse searched to target further papers in the initial screening. Full‐text review included examining the methods to ensure the paper used data for LMICs and evaluated results.

## 3 RESULTS

**FIGURE 1:**
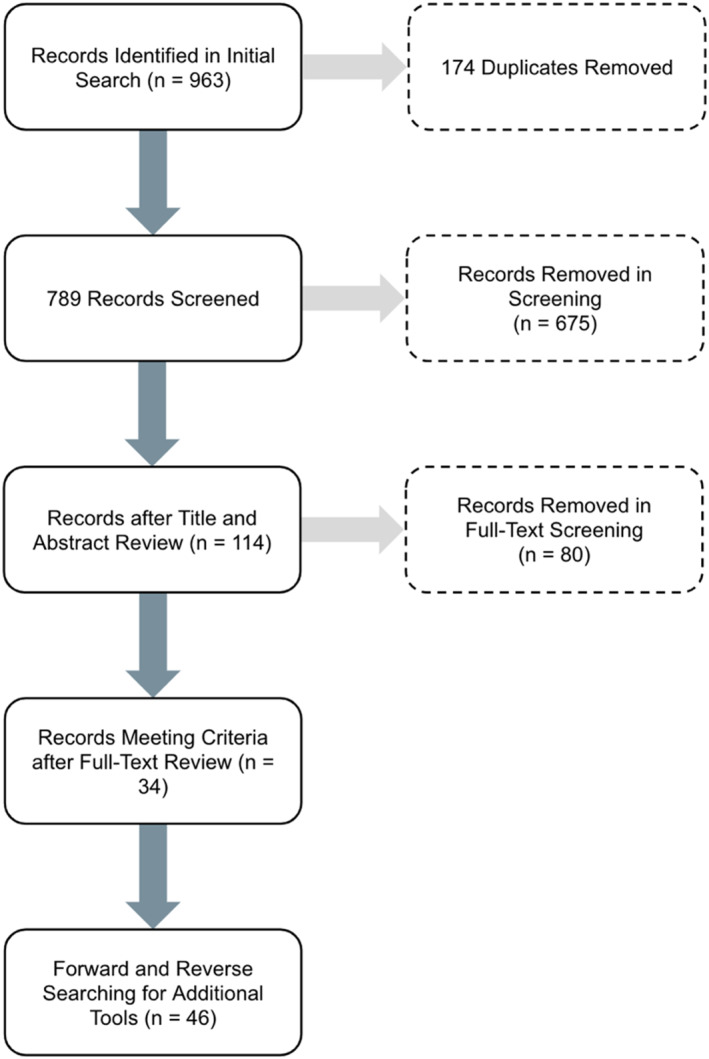
Overview of the scoping review.

The initial search yielded 963 papers and after 174 duplicates were removed, 789 papers underwent abstract and title screening as seen in Figure 1. The abstract and title screening removed 675 papers that did not meet describe global surgery assessment tools or showed multiple papers using the same tool. This left 114 papers for full‐text review. During full‐text review, 80 papers were removed because they did not meet inclusion criteria by either not describing a global surgery assessment tool or by repeating the use of a tool. This yielded 34 papers with unique global surgery assessment tools. Forward and reverse searching of full‐text review papers yield an additional 12 tools for a total of 46 tools.^9^, ^10^, ^11^, ^12^, ^13^, ^14^, ^15^, ^16^, ^17^, ^18^, ^19^, ^20^, ^21^, ^22^, ^23^, ^24^, ^25^, ^26^, ^27^, ^28^, ^29^, ^30^, ^31^, ^32^, ^33^, ^34^, ^35^, ^36^, ^37^, ^38^, ^39^, ^40^, ^41^, ^42^, ^43^, ^44^, ^45^, ^46^, ^47^, ^48^, ^49^, ^50^, ^51^, ^52^, ^53^, ^54^ Of these, 30 (65%) tools were quantitative, 1 (2%) qualitative, and 15 (33%) employed mixed‐methods. 25 (54%) tools evaluated surgery in general, whereas 21 (46%) were focused on various surgical subspecialties. All tools have been published and implemented.

Qualitatively, the analysis outlines several themes detailed in Table 1. We identified significant similarities among some tools. For instance, the Harvard Humanitarian Institute Tool, PIPES, and the WHO's Tool for Situational Analysis to Assess Emergency and Essential Surgical Care, while focusing on surgical capacity, have minor differences in analyzed variables and scope of their respective datasets. These differences aim to enhance the tool's applicability and effectiveness. PIPES, for example, reduced the number of variables to half that of the WHO's Tool for Situational Analysis to Assess Emergency and Essential Surgical Care to streamline its deployment. This underscores that the value of a tool is not entirely contingent on its novelty and improving upon existing tools can be valuable and potentially improve adoption.

**TABLE 1:** Identified themes and associated tools.

Theme	Tools addressing theme
Surgical capacity assessment and WHO framework alignment	Groen 2012: The PIPES tool^19^ LeBrun 2014: The harvard humanitarian institute tool^31^ Anderson 2017: Surgical volume and postoperative mortality rate at a referral hospital in western Uganda^12^ Anderson 2018: Assessment of capacity to meet lancet commission on global surgery indicators in the federal capital territory, Abuja, Nigeria^13^ Bari 2021: Surgical data strengthening in Ethiopia^16^ Joshipura 2006: WHO guidelines for essential trauma care that outline how to define a national trauma care system^47^
Tool development, methodologies, and organizational collaboration	Alexander 2019: Development and pilot testing of a context‐relevant safe anesthesia checklist for cesarean delivery in east Africa^10^ Hodges 2007: Questionnaire to examine anesthesia services in developing countries to understand surgical capacity and resources^36^ Merry 2010: Questionnaire surrounding the guidelines for safe anesthesia^37^ Notrica 2011: Survey aimed at identifying anesthesia and surgery needs by focusing on infrastructure^50^ Alidina 2019: Effectiveness of a multicomponent safe surgery intervention in Tanzania's lake zone^11^ Hayirli 2021: Development and content validation of the safe surgery organizational readiness tool^28^ Iverson 2020: Development of a surgical assessment tool for national policy monitoring & evaluation in Ethiopia^29^ Citron 2018: Surgical quality indicators in low‐resource settings: a new evidence‐based tool^24^ Ariyaratnam 2015: Toward a standard approach to measurement and reporting of perioperative mortality rate as a global indicator for surgery^15^ Roa 2020: Cross‐sectional study of surgical quality with a novel evidence‐based tool for low‐resource settings^40^ Chu 2010: Operative mortality in Médecins Sans Frontières surgical programs and associated factors^34^
Quantitative, qualitative measures, and data collection techniques	Bhashyam 2014: A novel approach for needs assessment to build global orthopedic surgical capacity in a low‐income country^18^ Iverson 2019: Mixed‐methods assessment of surgical capacity in two regions in Ethiopia^30^ Lin 2020: Identifying essential components of surgical care delivery through quality improvement^32^ Linden 2016: Community‐based questionnaire meant to identify surgical disease and determine access^48^ Groen 2012: Surgeons OverSeas assessment of surgical need (SOSAS) survey to identify surgical need and access^52^
Subspecialty focus	Ankomah 2015: Strategic assessment of the availability of pediatric trauma care equipment, technology, and supplies in Ghana^14^ Ploss 2017: Pilot use of a novel tool to assess neurosurgical capacity in Uganda^39^ Shapiro 2020: Development of a needs assessment tool to promote capacity building in hand surgery outreach trips^42^ Bola 2022: Assessment and validation of the community maternal danger score algorithm^21^ Beyene 2021: Cesarean delivery rates, hospital readiness, and quality of clinical management in Ethiopia: National results from two cross‐sectional emergency obstetric and newborn care assessments^17^ Botelho 2021: Implementation of a checklist to improve pediatric trauma assessment quality in a brazilian hospital^46^ Cairo 2018: Pedi‐PIPES: Characterizing pediatric surgical capacity in the eastern democratic republic of Congo^22^ Zha 2021: Assessment of anesthesia capacity in public surgical hospitals in Guatemala^45^ Wong 2014: The international assessment of capacity for trauma (INTACT): An index for trauma capacity in low‐income countries^20^ Sandie 2023: Study examining emergency c‐section and elective C‐section rates to determine access to surgery^33^ Forcillo 2019: Cardiac surgery assessment tool piloted in three countries^43^
Geospatial analysis, disease prevalence, and system assessments	Cairo 2020: GAPS: Geospatial mapping of pediatric surgical capacity in north Kivu, democratic republic of Congo^23^ van Kesteren 2022: PREvalence study on surgical COnditions (PRESSCO) 2020: A population‐based cross‐sectional countrywide survey on surgical conditions in post‐ebola outbreak Sierra Leone^44^ Cotache‐Condor 2021: Geospatial analysis of pediatric surgical need and geographical access to care in somaliland^26^ Prin 2018: Using emergency‐to‐elective ratio to better understand surgical capacity, need, and access^53^ Samad 2015: Study focused on screening in the community to measure access to and need for surgery in Pakistan^49^ Dahir 2020: Interpreting the lancet surgical indicators in somaliland^27^ Albutt 2019: A nationwide mixed‐methods evaluation of private sector surgical capacity in Uganda^9^ Walker 2010: Cross‐sectional survey to identify key factors for pediatric surgery capacity and quality^54^
Evaluation and impact assessment of surgical tools	Odland 2022: Identifying a basket of surgical procedures to standardize global surgical metrics^38^ Equi‐trauma collaborative 2022: Defines the four‐delay framework to understand barriers to accessing quality surgical care^25^ Bowman 2013: Examines 32 pediatric surgical procedures across countries to determine capacity and limitations^35^ Ravi 2022: Optimal resources for children’ surgery tool to identify gaps in surgical care and examine criteria compliance^41^ Whitaker 2021: Three delays framework identifies barriers toward improving surgical care and aims toward a new methodology^51^

The widespread use of checklists in identified tools is notable. They permit a quantifiable surgical assessment while also ensuring ease of implementation across varied global settings. Despite their simplicity and accessibility, checklists are also limited in some keyways. Although they offer value in comparing a single system at various timepoints, comparing multiple systems in different regions may not address confounding variables and obscure rather than illuminate, legitimate similarities, or differences. Although tools, such as the WHO Surgical Assessment Tool (SAT), are designed for global comparison based on broadly comparable metrics, others, such as international assessment of capacity for trauma and pediPIPES, aim to evaluate specific systems, reflecting the diverse needs of surgical assessment.^20^, ^22^, ^27^ Similarly, they are limited and a dysfunctional OR or health system may appear effective on a checklist that does not account for challenging qualitative factors.

Identifying key variables that can reliably represent surgical capacity helps minimize the work, time, and funding required for surgical assessment. To accomplish this, some tools use proxy variables where they can measure one or a few key factors and estimate surgical capacity.^14^, ^15^ Direct observation also emerged as a notable qualitative assessment method, offering validated insights, despite depending on the observer perspective. To combat this, the inclusion of multiple observers enhanced validity overall.

In our assessment of the broader categories that encompass each tool, many tools were observed to be surgical subspecialty‐focused, reflecting the diverse requirements within surgical care. Prominently, tools aligning with WHO frameworks, such as the Harvard Humanitarian Institute Tool and WHO's Situational Analysis Tool, illustrate the synergy between global standards and individual tool design. We also discuss the use of quantitative and qualitative measures in data collection, highlighting the need for adaptable assessment strategies. The application of geospatial analysis and the assessment of disease prevalence emphasize the critical role of data in pinpointing and understanding surgical needs across different regions. Moreover, the evaluation and impact assessment of these tools are pivotal in enhancing global surgical practices, offering essential insights for policy‐making, identifying areas for improvement, and tailoring surgical interventions to meet diverse population needs. These themes collectively underscore the complex multifaceted nature of global surgery assessment, vital for the effective evaluation and enhancement of worldwide surgical systems.

## 4 DISCUSSION

Since the recognition of global surgery as a field in academia, there has been a proliferation of surgical assessment tools, predominantly focusing on nonspecialty surgeries. A new dynamic evolved in this landscape. There was an early apparent surplus of tools for general surgery assessment, particularly relevant in LMICs where general surgeries are more commonly required and more readily available. This proliferation is likely due to the higher frequency of general surgery programs and the pressing need in these regions. In total, the number of published tools for all specialties total and general surgery is roughly equal. However, there remains a striking scarcity of assessment tools for several surgical subspecialties, including but not limited to vascular surgery, thoracic surgery, and otolaryngology. This gap is noteworthy considering that many of the existing tools for general surgery assessment such as PIPES, Harvard Humanitarian Institute Tool (HHI), and WHO SAT often share similar assessment features.^19^, ^27^, ^31^

Lin et al. (2020) aimed to synthesize existing guidelines and create a unified SAT through a Delphi method and WHO vetting process.^55^ Their updated tool consists of 169 items spanning the following five domains: infrastructure, service delivery, workforce, information management, and financing. This tool is designed to be universally applicable, facilitating systematic assessments at local, national, and international levels.

However, our review focuses on identifying and categorizing a wide range of existing assessment tools, emphasizing the diversity in their design and application. Although Lin et al. provide a new standardized tool, we believe that there is a need for a broader array of tools tailored to specific contexts and surgical subspecialties. We emphasize the importance of local leadership and involvement in the development and implementation of surgical assessment tools. This aligns with the broader goals of the UN Sustainable Development Goals, which emphasize robust monitoring and accountability in healthcare services. By including local stakeholders, assessment tools can be better adapted to the specific needs and contexts of LMICs, enhancing their relevance and effectiveness.

There has been a notable expansion into tools tailored for specific surgical subspecialties, such as ophthalmology, pediatric surgery, and plastic surgery, among others. Certain specialty fields are more represented in global surgery, such as plastic surgery, whereas others, such as cardiac surgery and transplantation, remain more neglected. This is possibly due to a perceived relative increased need for these specialties in these settings. For example, pediatric surgery is a focus because in many LIMCs, the average age is under 18.^56^ The relative ease of establishing these specialty surgery programs is also considered. Most general plastic surgery does not require the use of complex equipment such as cardiopulmonary bypass or need a detailed policy basis such as transplantation. Finally, there is a difference in the availability of surgeons trained in these specialties. There are far more general surgeons globally than any subspecialty, and smaller specialties such as cardiac and transplantation will naturally be less represented. These specialized tools, along with those designed for anesthesia—a crucial element in the delivery of three of the five phases of surgical care—highlight the sector's evolution and adaptability. This review further identifies aspects of surgery that can be performed by many general surgeons that are neglected such as wound care, thoracic injuries, and postoperative infection management.

The methodology behind the development of these tools is as crucial as their purpose. Different methodologies, whether qualitative, quantitative, or mixed‐methods, bring unique strengths. Although mixed‐methods offer a comprehensive approach, they are not inherently superior to well‐executed qualitative or quantitative methods aimed at addressing specific research questions. The choice of methodology often dictates the tool's effectiveness, reach, and adaptability across diverse healthcare systems and geographical regions. Geospatial analysis and disease prevalence assessments are other vital components of a comprehensive assessment approach. By understanding geographical disparities and disease patterns, health systems can more effectively allocate resources and plan surgical services to meet the specific needs of different areas.

In this paper, we categorize the tools by broad categories and emphasize the differences and similarities of the tools (Table 2). Surgical assessment should not be performed as a one‐size‐fits‐all approach, and the diversity of tools highlighted demonstrate that the best specific tool should be used for the appropriate context. Although some tools objectively offer more information, such as a database metric tool, it may not be feasible to apply that tool in a center that does not have an accurate database management. Tools also have different priorities; some tools are used for locally‐based improvement, while others align with national and international priorities, such as the National Surgical Obstetric and Anesthesia Plan based tools. This paper provides a collection of effectively implemented surgical assessment tools that may be further analyzed in different ways to highlight different aspects of the tools. For example, many of the tools analyzed heavily focus on the intraoperative phase of surgery and there may be an opportunity to better describe the other stages of surgery. Other analyses could use this review of global surgery assessment tools to identify further gaps in the literature and guide further research.

**TABLE 2:** Global surgery assessment tools.

Tool	Citation	Qualitative/Quantitative/Mixed‐methods	Specialty versus nonspecialty	Description of tool	Number of citations
Surgical assessment tool with qualitative interviews^9^	Albutt K, Drevin G, Yorlets RR, et al. 2019	Mixed‐methods	Nonspecialty	Nationwide mixed‐methods evaluation of private and private‐not‐for‐profit surgical capacity in Uganda, revealing significant barriers such as workforce shortages, high medical and nonmedical costs, and poor coordination of care, which impede the delivery of effective and timely surgical services in these sectors	7
“Safe cesarean delivery checklist” with clinical observations^10^	Alexander LA, Newton MW, McEvoy KG, et al. 2019	Mixed‐methods	Specialty‐focused (anesthesia)	Authors developed and piloted an obstetric anesthesia checklist for cesarean delivery in East Africa, significantly improving adherence to best practice guidelines during simulated obstetric emergencies	14
Surgical safety checklist with qualitative interviews^11^	Alidina S, Kuchukhidze S, Menon G, et al. 2019	Mixed‐methods	Nonspecialty	Evaluated the effectiveness of the safe surgery 2020 intervention in Tanzania, aimed at improving surgical quality through a multicomponent strategy focused on leadership, safe practices, sterilization, data quality, and infrastructure	26
Ugandan case review with OR observation^12^	Anderson GA, Ilcisin L, Abesiga L, et al. 2017	Mixed‐methods	Nonspecialty	This study assessed surgical volume and postoperative mortality at a referral hospital in Western Uganda, demonstrating that logbooks are an effective method for collecting these data in low‐resource settings	47
Nigeria NSOAP preparation plan^13^	Anderson JE, Ndajiwo AB, Nuhu SA et al. 2018	Quantitative	Non‐specialty	This baseline assessment of surgical capacity in Nigeria's federal capital territory revealed that although most patients can access hospitals within 2 h, there are significant deficiencies in SAO provider density, surgical volume, and perioperative mortality rate tracking	13
Assessment of pediatric trauma care tools as proxy for peds care with qualitative interviews^14^	Ankomah J, Stewart BT, Oppong‐Nketia V, et al. 2015	Mixed‐methods	Specialty (pediatric trauma)	The study assessed the availability of pediatric trauma care items in Ghana, finding significant deficiencies due to equipment absence, lack of training, stock‐outs, and technology breakage, with pediatric items consistently less available than adult‐sized items	46
Post‐operative mortality rate as a proxy for surgical safety^15^	Ariyaratnam R, Palmqvist CL, Hider P, et al. 2015	Quantitative	Non‐specialty	This study evaluated the perioperative mortality rate (POMR) as a global indicator of surgical safety across different income countries, highlighting the need for standardized measurement and risk adjustment to improve its accuracy and usefulness	68
Kirkpatrick method with SaLTs key performance indicators^16^	Bari S, Incorvia J, Iverson KR, et al. 2021	Mixed‐methods	Non‐specialty	This study demonstrates the successful implementation of a data quality intervention in Ethiopian hospitals, improving surgical data collection and reporting through enhanced teamwork, communication, and accountability despite persistent barriers including inadequate human resources and limited technological infrastructure	17
National cross‐sectional surveys or assessments of emergency obstetric and newborn care (EmONC)^17^	Beyene MG, Zemedu TG, Gebregiorgis AH, et al. 2021	Quantitative	Specialty (OBGyn)	The study assessed cesarean delivery rates, hospital readiness, and quality of clinical management in Ethiopia using two national cross‐sectional emergency obstetric and newborn care assessments, highlighting improvements in service availability and aspects of clinical care between 2008 and 2016	12
Orthopedic surgery audience response conference questionnaire^18^	Bhashyam AR, Fils J, Lowell J, et al. 2014	Quantitative	Specialty (orthopedic surgery)	The study used an electronic audience response system (ARS) during a Haitian continuing medical education conference to successfully improve response rates and better assess the needs of local orthopedic surgeons, identifying key areas for knowledge and skill enhancement	25
PIPES^19^	Groen RS, Kamara TB, Dixon‐Cole R, et al. 2012	Quantitative	Non‐specialty	The PIPES tool, developed by surgeons OverSeas (SOS), was used to assess and document surgical capacity in Sierra Leone's government hospitals by evaluating personnel, infrastructure, procedures, equipment, and supplies, showing significant improvements in surgical capacity between 2008 and 2011	109
INTACT^20^	Wong EG, Gupta S, Deckelbaum DL, et al. 2014		Specialty (trauma)	The INTACT index is a standardized tool designed to assess a healthcare facility's trauma care capacity, evaluating 40 key elements and revealing significant deficiencies in personnel, imaging, fracture repair, and burn management in Sierra Leone's hospitals	55
Community maternal danger score^21^	Bola R, Ujoh F, Ukah UV, et al. 2022	Quantitative	Specialty (OBGyn)	The community maternal danger score (CMDS) is a low‐cost evidence‐based tool using seven risk factors to identify pregnant women needing a skilled birth attendant, validated through analysis of medical records in Nigeria and showing good predictive ability for the need for skilled care and in‐hospital mortality	5
Pedi‐PIPES^22^	Cairo SB, Kalisya LM, Bigabwa R, et al. 2018	Quantitative	Specialty (pediatric surgery)	The pediatric personnel, infrastructure, procedures, equipment, and supplies (PediPIPES) survey was used to assess pediatric surgical capacity in the eastern democratic republic of Congo, revealing significant deficiencies in personnel and infrastructure compared to other sub‐Saharan African countries, with most hospitals lacking pediatric surgeons and reliable utilities	14
Global assessment of pediatric surgery tool (GAPS) with geospatial mapping^23^	Cairo SB, Pu Q, Malemo Kalisya L, et al. 2020	Quantitative	Specialty (pediatric surgery)	The global assessment of pediatric surgery tool was used to analyze geographic variability and access to pediatric surgical services in North Kivu, DRC, revealing significant limitations in resources, trained providers, and access to critical care, highlighting the need for strategic improvements in healthcare delivery	12
Surgical quality indicators tool^24^	Citron I, Saluja S, Amundson J, et al. 2018	Quantitative	Non‐specialty	The novel tool provides an evidence‐based framework with 15 quality indicators to assess and improve the quality of surgical care in low‐resource settings, encompassing safe, effective, patient‐centered, timely, efficient, and equitable dimensions of care	21
Four‐delay framework with WHO building blocks and institution of medicine quality outcomes frameworks^25^	Equi‐Trauma Collaborative, Odland ML, Abdul‐Latif AM, et al. 2022	Mixed‐methods	Specialty (trauma care)	The study used a mixed‐methods approach to identify 121 barriers to accessing quality injury care in Ghana, South Africa, and Rwanda, finding that 58% of common barriers were related to delays in receiving quality care, highlighting the need for context‐specific solutions and multiple data collection methods to effectively address these barriers	18
SOSAS+ WHO's (WHO) surgical assessment tool‐hospital Walkthrough and the global initiative for Children's surgery global assessment in pediatric surgery^26^	Cotache‐Condor CF, Moody K, Concepcion T, et al. 2021	Mixed‐methods	Specialty (pediatric surgery)	Using geospatial analysis, this study assessed the geographical distribution and access to pediatric surgical care in Somaliland, finding that less than 10% of children have timely access to care, with significant disparities particularly affecting those in rural areas where delays can exceed 3 years	9
WHO surgical assessment tool using lancet commission on global surgery indicators in Somaliland^27^	Dahir S, Cotache‐Condor CF, Concepcion T, et al. 2020	Mixed‐methods	Non‐specialty	This study used the lancet commission on global surgery (LCoGS) indicators to evaluate Somaliland's surgical health system, revealing significant gaps with only 19% of the population having timely access to essential surgery, a surgical workforce density of 0.8 SAO providers per 100,000 people, and a surgical volume of 368 procedures per 100,000 people, all well below LCoGS goals	18
Safe surgery organizational readiness tool^28^	Hayirli TC, Meara JG, Barash D, et al. 2021	Quantitative	Non‐specialty	The safe surgery organizational readiness tool is a validated instrument with 14 domains and 56 items designed to assess the readiness of surgical facilities in low‐ and middle‐income countries to implement surgical safety and quality improvement interventions by evaluating individual, team, and facility‐level beliefs and attitudes	7
WHO service availability and readiness tool (SARA)^29^	Iverson KR, Ahearn O, Citron I, et al. 2020	Quantitative	Non‐specialty	The adapted WHO tool for situational analysis (SaLTS) was used to assess surgical, obstetric, and anesthesia capacity in Ethiopia, revealing that whereas facilities met a significant portion of both international and national benchmarks, there were notable discrepancies, particularly in specialized surgeries, highlighting the need for tools that balance global standards with national‐specific targets	11
Lancet commission tools: Hospital assessment tool with qualitative interviews^30^	Iverson KR, Garringer K, Ahearn O, et al. 2019	Mixed‐methods	Non‐specialty	The study used a mixed‐methods approach to assess surgical capacity in two Ethiopian regions, finding significant deficiencies in service delivery, infrastructure, workforce, information management, and financing, highlighting the need for comprehensive improvements to enhance surgical, anesthesia, and obstetric services	20
Harvard humanitarian institute tool^31^	LeBrun DG, Chackungal S, Chao TE, et al. 2014	Quantitative	Non‐specialty	The Harvard humanitarian initiative survey tool assessed 78 district hospitals in seven low‐ and middle‐income countries, revealing significant gaps in surgical infrastructure, limited availability of essential equipment and medicines, and a shortage of surgeons and anesthesiologists, underscoring the need to prioritize essential surgery and safe anesthesia in global health agendas	143
2020 updated surgery tool from harvard based on a brief review‐PGSSC‐SAT^32^	Lin Y, Raykar NP, Saluja S, et al. 2020	Quantitative	Non‐specialty	The surgical assessment tool, developed through a systematic review, expert opinion, and world health organization vetting, aggregates 169 essential items across infrastructure, service delivery, workforce, information management, and financing, providing a comprehensive framework for assessing and scaling up surgical systems globally	10
Service provision assessment from DHS^33^	Sandie AB, Mutua MK, Sidze E, et al. 2023	Quantitative	Non‐specialty	The study used demographic and health surveys data from 21 sub‐Saharan African countries to evaluate the prevalence of elective and emergency caesarean sections, identifying that emergency CS (4.6%) is more common than elective CS (3.4%), and associated socioeconomic factors, finding higher prevalence in private facilities and among wealthier women, with emergency CS linked to increased early neonatal mortality, highlighting the need for improved quality of antenatal and postnatal care	2
Médecins sans frontiers data analysis^34^	Chu KM, Ford N, Trelles M. 2010	Quantitative	Non‐specialty	The study evaluated operative mortality in Médecins Sans Frontières surgical programs across 13 resource‐limited countries, revealing an overall low operative mortality rate of 0.2%, with higher risk associated with conflict settings, emergency procedures, and specific surgeries, emphasizing the feasibility of safe surgical care with proper standards and protocols in these environments	74
Pediatric surgery assessment tool^35^	Bowman KG, Jovic G, Rangel S, et al. 2013	Quantitative	Specialty (pediatric surgery)	The study utilized a modified WHO instrument to assess the availability and safety of 32 essential and emergency pediatric surgical procedures across 103 hospitals in Zambia, finding that surgical skills were the primary limitation in providing care, with minimum safety standards met by only 14% of hospitals	48
Anesthesia services in developing countries: Defining the problems‐ anesthesia questionnaire^36^	Hodges SC, Mijumbi C, Okello M, et al. 2007	Quantitative	Specialty (anesthesia)	A questionnaire was used to identify the challenges in providing anesthesia in Uganda, revealing that only 23% of anesthetists can deliver safe anesthesia to adults, 13% to children, and 6% for caesarean sections, highlighting critical shortages in personnel, drugs, equipment, and training, and underscoring the need for substantial investment to improve anesthesia safety and services	400
Anesthesia recommended questionnaire^37^	Merry AF, Cooper JB, Soyannwo O, et al. 2010	Quantitative	Specialty (anesthesia)	The international standards for a safe practice of anesthesia, established by the world federation of societies of anesthesiologists (WFSA) and adopted in 2010, provide a comprehensive set of guidelines aimed at improving the quality and safety of anesthesia care globally, with specific recommendations for different resource settings, emphasizing the continuous presence of a qualified anesthesia professional, proper training and certification, the use of monitoring devices, and adherence to safety checklists and protocols	322
“Surgical basket” 32 procedures to test for capacity^38^	Odland ML, Nepogodiev D, Morton D, et al. 2021	Quantitative	Non‐specialty	The study defined a globally applicable list of 32 surgical procedures through a Delphi exercise, aimed at standardizing the assessment of surgical capacity across health systems, to facilitate better monitoring and evaluation of global surgical care	15
NeuroPIPES^39^	Ploss B, Abdelgadir J, Smith ER, et al. 2017	Quantitative	Specialty (neurosurgery)	The NeuroPIPES tool, adapted from the surgeons OverSeas PIPES assessment, was piloted to evaluate neurosurgical capacity at three public hospitals in Uganda, revealing variations in scores that corresponded with the number of neurosurgeons and caseloads, but also highlighting the need for further refinement to improve reliability and validity	16
Amazon assessment tool‐adapted from the cintron tool^40^	Roa L, Citron I, Ramos JA, et al. 2020	Quantitative	Non‐specialty	The study successfully applied a novel tool to measure surgical quality in a resource‐limited tertiary hospital in Amazonas, Brazil, demonstrating feasibility by collecting all 14 quality metrics and achieving proposed targets for timely processes and effective outcomes, while highlighting areas for further refinement to enhance surgical quality improvement	8
Global initiative for Children's surgery (GICS) group produced the optimal resources for Children's surgery (OReCS)^41^	Ravi K, Killen A, Alexander A, et al. 2022	Quantitative	Specialty (pediatric surgery)	The OReCS audit tool, implemented across 10 hospitals in eight countries, successfully identified gaps in children's surgical care by measuring essential criteria compliance and suggesting improvements for local and national surgical plans, with feedback indicating its practicality across diverse settings	2
Hand surgery checklist and qualitative paper^42^	Shapiro LM, Park MO, Mariano DJ, et al. 2020	Mixed‐methods	Specialty (hand surgery)	The study developed a standardized needs assessment tool tailored for hand surgery outreach trips, incorporating seven domains—Human resources, physical resources, procedures, cultural and language barriers, safety, quality and access, regulation and cost, and knowledge transfer and teaching—To ensure effective site selection, resource allocation, and delivery of high‐quality care in low‐ and middle‐income countries	12
Cardiac surgery assessment tool^43^	Forcillo J, Watkins DA, Brooks A, et al. 2019	Mixed‐methods	Specialty (cardiac surgery)	The study identified key variables for a cardiac surgery needs assessment tool by evaluating current initiatives, challenges, and opportunities at cardiac surgery centers in Namibia, Zambia, and Uganda, revealing adequate facilities and surgical expertise but significant gaps in support staff, material resources, comprehensive care, and access for remote patients	40
The PREvalence study on surgical COnditions (PRESSCO)^44^	van Kesteren J, van Duinen AJ, Marah F, et al. 2022	Mixed‐methods	Non‐specialty	The PREvalence study on surgical COnditions (PRESSCO) 2020 used an expanded version of the SOSAS tool to estimate the national prevalence of conditions requiring surgical consultation in Sierra Leone, finding a significant reduction in such conditions compared to 2012 despite disruptions from the Ebola epidemic, with financial constraints being the main barrier to seeking care	3
World federation of societies of anesthesiologists (WFSA) anesthesia facility assessment tool^45^	Zha Y, Truché P, Izquierdo E, et al. 2021	Mixed‐methods	Specialty (anesthesiology)	Using the WFSA anesthesia facility assessment tool, a national survey of Guatemalan public hospitals in 2018 revealed significant deficiencies in anesthesia care capacity, including inadequate personnel, essential medications, and equipment, highlighting the need for policy initiatives to improve safe anesthesia and surgical care	8
Brazil hospital introduces checklist^46^	Botelho F, Truché P, Caddell L, et al. 2021	Quantitative	Non‐specialty	The study aimed to evaluate adherence to standardized trauma care for children following the introduction of a checklist in a busy Brazilian trauma center, finding no significant improvement in protocol adherence or clinical outcomes post‐intervention, indicating a need for enhanced pediatric trauma education and organized triage approaches	3
WHO guideline for essential trauma care^47^	Joshipura M. 2006	Quantitative	Specialty (trauma)	The guidelines for essential trauma care have facilitated the development of a national trauma care system in India, emphasizing systematic changes, improving advocacy, and highlighting the need for a national policy for trauma care	40
Rwandan community‐based survey assessing care for nonobstetric surgical conditions including interview questionnaire with physical exams^48^	Linden AF, Maine RG, Hedt‐Gauthier BL, et al. 2016	Qualitative	Non‐specialty	This study validated a community‐based questionnaire in Burera district, Rwanda, to accurately identify untreated surgically correctable conditions and found it had high specificity but low sensitivity, highlighting the importance of accurate surveys in planning surgical services	12
Indus hospital cohort screening questionnaire^49^	Samad L, Jawed F, Sajun SZ, et al. 2015	Quantitative	Non‐specialty	This study in Karachi, Pakistan found a high prevalence of symptoms requiring surgical assessment, but low follow‐up rates for scheduled appointments, highlighting the need for a validated tool to identify surgical diseases and understand health‐seeking behavior	0
Rwandan hospital questionnaire^50^	Notrica MR, Evans FM, Knowlton LM, et al. 2011	Quantitative	Non‐specialty	This survey revealed significant deficiencies in surgical and anesthesia infrastructure at district hospitals in Rwanda, including limited access to oxygen, anesthesia equipment, medications, and trained personnel, highlighting the need for continued development of emergency and essential surgical services to improve healthcare	138
Three delays framework^51^	Whitaker J, O'Donohoe N, Denning M, et al. 2021	Quantitative	Specialty (trauma)	This systematic review of LMIC trauma systems assessments using the three delays framework revealed that the majority of studies focused on barriers to receiving care (delay 3), with fewer studies addressing barriers to seeking (delay 1) and reaching care (delay 2), and that many methodologies could be adapted for rapid assessment	47
Surgeons OverSeas assessment of surgical need (SOSAS)^52^	Groen RS, Samai M, Stewart KA, et al. 2012	Mixed‐methods	Non‐specialty	The surgeons OverSeas assessment of surgical need (SOSAS) survey conducted in Sierra Leone found that 25% of respondents required surgical care and 25% of deaths in the previous year could have potentially been prevented with timely surgical intervention, highlighting a significant unmet need for surgical services in low‐income settings	249
Emergency‐to‐elective ratio^53^	Prin M, Guglielminotti J, Mtalimanja O, et al. 2018	Quantitative	Non‐specialty	The emergency‐to‐elective surgery ratio was created after a systematic review of surgical volume and acuity from 2006 to 2016. Low‐ and middle‐income countries had significantly higher ratios of emergency surgeries and were seen as having less access to elective surgical services. The emergency‐to‐elective ratio was seen as a valid and simple indicator of access to surgical care	57
Pediatric surgery and anesthesia in Uganda: Cross‐sectional survey^54^	Walker IA, Obua AD, Mouton F, 2010	Quantitative	Specialty‐Pediatrics	This study described a cross‐sectional survey tool used to quantify key factors of pediatric surgery including mortality, surgical rate, and number of surgeons. It was used in Uganda and has been effective in assessing access and quality	111

Finally, the continuous evaluation and impact assessment of these tools are imperative for their improvement and effectiveness. This process involves assessing not only the tools themselves but also their influence on surgical care delivery, health outcomes, and policy‐making processes. This process was described in several tools.^41^ A comprehensive, multifaceted approach is essential in the development and implementation of surgical assessment tools, especially for global surgery.^57^ Such an approach ensures that these tools are not only technically robust but also attuned to the diverse and specific needs of surgical systems worldwide, with a particular focus on LMICs. Furthermore, the level of ownership and local leadership of the tools is difficult to capture in a review. To protect against neocolonial influence of high‐income countries, the most effective tools should be locally led and created with the intention of local staff leading the initiatives. This holistic view is key to advancing the field of global surgery, enhancing health outcomes, and informing effective policy‐making.

### 4.1 Limitations

This review only reviewed academically published global surgery assessment tools and did not include tools that may have been published in other languages or in journals not indexed on PubMed. This excludes many tools that have been used successfully by nongovernmental organizations, governmental and intergovernmental agencies, or hospitals but have not recorded their results. This paper further only analyzed parameters such as quantitative, qualitative, and mixed‐methods, specialty versus nonspecialty, and number of citations. Further analysis could investigate countries analyzed, data sample sizes, phases of surgery highlighted, local government support or buy‐in of the tools, and article downloads could provide further insights.

## 5 CONCLUSIONS

The field of academic global surgery has produced many global surgery assessment tools designed for use in LMICs. Many of the tools share common characteristics, and the different methodologies of the tools provide different strengths and weaknesses for different contexts. This paper creates a collection of effectively implemented global surgery assessment tools and provides a valuable resource for researchers in the field to analyze when deciding which SAT to use and to consult in the design of new surgical assessment tools. We hope that this paper will help future researchers minimize overlap and maximize effectiveness in their design of further global surgery assessment tools in much needed areas such as thoracic surgery, postoperative care, and wound care. Ideally, these tools should be designed by or with local surgical leadership. Further efforts should focus on expanding assessment tools for neglected specialties and expanding the use of validated established tools so that there can be an accurate comparison across groups or time courses.

## AUTHOR CONTRIBUTIONS

**Kevin Gianaris**: Formal analysis; investigation; methodology; writing—original draft; writing—review & editing. **Brooke Stephanian**: Formal analysis; investigation; methodology; writing—original draft; writing—review & editing. **Sabin Karki**: Formal analysis; investigation; methodology; writing—original draft; writing—review & editing. **Shailvi Gupta**: Project administration; writing—review & editing. **Amila Ratnayake**: Project administration; writing—review & editing. **Adam L. Kushner**: Project administration; writing—review & editing. **Reinou S. Groen**: Project administration; writing—review & editing.

## CONFLICT OF INTEREST STATEMENT

The authors declare that they have no conflicts of interest.

## ETHICS STATEMENT

This research did not involve human or animal subjects. This study complied with all institutional ethical requirements.
